# Sexual Double Standard: A Psychometric Study From a Macropsychological Perspective Among the Spanish Heterosexual Population

**DOI:** 10.3389/fpsyg.2019.01869

**Published:** 2019-08-14

**Authors:** María del Carmen Gómez Berrocal, Pablo Vallejo-Medina, Nieves Moyano, Juan Carlos Sierra

**Affiliations:** ^1^Mind, Brain, and Behavior Research Center (CIMCYC), University of Granada, Granada, Spain; ^2^SexLab KL, Fundación Universitaria Konrad Lorenz, Bogotá, Colombia; ^3^Faculty of Human Sciences and Education, University of Zaragoza, Huesca, Spain

**Keywords:** sexual double standard, macropsychological indicator, heterosexual relationships, psychometric properties, sexual freedom, sexual shyness

## Abstract

In the context of heterosexual relationships, the sexual double standard (SDS) leads to a more negative assessment of women than men when they exhibit the same sexual behavior. This work assumes that in Western democratic societies, the individual attitude toward SDS takes different forms due to the processes of conviction regarding the social norm that exists on this matter. Therefore, the individual attitude toward SDS will depend on the person’s perception of what others think about that topic. We distinguish between self-referred response, it refers to subjects’ personal endorsement of the SDS, and responses hetero-referred, subjects’ perception of sexual societal double standard. This paper presents a version of the Double Standard Scale (SDSS) that assesses the subjective perception of society’s support for the SDS. We examine its psychometric properties in a sample of Spanish population heterosexual of 1,206 individuals (50% males), distributed across three groups (18–34, 35–49, and 50 years old or older). We performed Exploratory and Confirmatory Factor Analysis. The final version consists of 18 item distributed into three factors (Acceptance for Male Sexual Shyness, Acceptance for Female Sexual Freedom and Acceptance for Traditional Double Standard). Said three-factor structure does not match with the two-factor structure of the self-referred form. Internal consistency, temporal stability and validity evidence are reported. This version of the SDSS is reliable and valid. No gender differences are found in the SDSS-H. However, the results show that the context of group membership, based on education and age, is differently associated with the response to SDSS-H. That is, higher scores are found for individuals with the highest education and for the youngest participants. We discuss the usefulness of this measure to improve the prediction of individual attitude toward SDS, as well as, to evaluate the SDS phenomenon at a level of macropsychological analysis.

## Introduction

The sexual double standard (SDS) consists of judging heterosexual men and women differently for the same sexual behavior. The traditional SDS implies that men always think about sex and women must be “gate-keepers” of their own sexuality (Seabrook et al., 2016). As a result, the traditional SDS favors highly sexually active heterosexual men to be evaluated more positively than women who exhibit the same behavior (Zaikman and Marks, 2017). The assessment of the prevalence of SDS is relevant for understanding sexual health, which is related to the ability to freely enjoy and express sexuality (Lottes, 2000). In accordance with this, SDS has been associated with several sexual related issues, such as sexual victimization (Sierra et al., 2011; Eaton and Matamala, 2014) sexual assaults (Sierra et al., 2009; Bliss, 2014; Eaton and Matamala, 2014; Moyano et al., 2017), victim-blaming attitudes (Gracia et al., 2018), higher risk of acquiring sexually transmitted infections (Bermúdez et al., 2013; Fasula et al., 2014; Ramiro-Sánchez et al., 2018) and lower sexual satisfaction (Haavio-Mannila and Kontula, 2003; Santos-Iglesias et al., 2009). There are data that support the presence of SDS in Spanish society (Gutiérrez-Quintanilla et al., 2010; Mascheroni et al., 2015) as well as the existence of differential traditional sexual schemes for men and women, for example, in the field of sexual pleasure (López-Sáez et al., 2008) or sexual satisfaction (Sánchez-Fuentes and Santos-Iglesias, 2016). Therefore, the study of SDS in this cultural context continues to be relevant.

Sexual liberation has gradually contributed to the manifestation of traditional SDS to be expressed in a more subtle way in some fields. For example, premarital sex is increasingly accepted in men and women (Wells and Twenge, 2005). However, the age of first sexual intercourse of men is lower than that of women (Ortiz et al., 2011; Peixoto et al., 2016) and men have more number of sexual partners (Chi et al., 2015; Sánchez-Fuentes et al., 2016). In addition, still some mass media and exposure to certain games strength this image of stereotyped males and females, which often reinforces sexism among youngsters (Bègue et al., 2017). In other words, the SDS seems a pervasive phenomenon in society, but the empirical evidence is inconsistent. On the one hand, while studies using standardized questionnaires provide evidence of the double sexual standard (Crawford and Popp, 2003), studies that use a person perception task rarely show that people evaluate men and women differently based on their sexual activity (Marks and Fraley, 2005).

To explain the difficulty of assessing the SDS, it has been pointed out that many empirical studies on the topic have not considered certain methodological aspects (e.g., Crawford and Popp, 2003; Wells and Twenge, 2005; Sagebin and Sperb, 2013). It is also suggested that researchers should take a more theoretical approach to understanding the SDS (Zaikman and Marks, 2017).

### The Assessment of the Prevalence of Sexual Double Standard: Methodological Limitations

A possible cause for the inconsistency observed in the prevalence of the SDS is that results obtained with different designs are sometimes used to determine the incidence of this phenomenon. In within-subject designs, participants respond to the same questions for each target (targets: men and women); thus, the direct response of the subject yields a measure of individual endorsement of the SDS (Crawford and Popp, 2003). By contrast, in between-subject designs each participant assesses a single target (either a man or a woman). In this case, the direct response of each subject yields a measure of individual acceptance of certain sexual behaviors in men or in women (Crawford and Popp, 2003). As a result, each type of design is useful to determine the incidence of an attitude toward different topics. For this reason, Sakaluk and Milhausen (2012) recommended using appropriate study designs to capture such differences.

On the other hand, sometimes the items of the standardized instruments used to measure the SDS may assess individuals’ attitude toward different topics (e.g., attitude toward the SDS vs. attitude toward sexual behaviors in men and women). For example, according to Sagebin and Sperb (2013), the Sexual Double Standard Scale (SDSS, Muehlenhard and Quackenbush, 2011) measures the attitudes of subjects toward both the SDS (e.g., “Women are naturally more monogamous – inclined to stick with one partner – than are men”) and certain sexual behaviors in men and women (e.g., “A woman should be sexually experienced when she gets married”). The same diversity is found in the items of the Scale for the Assessment of Sexual Standards among Youth (SASSY, Emmerink et al., 2017); five of their nineteen items assess the attitude of the respondent toward some sexual behaviors either in boys or girls (e.g., “Sometimes a boy should apply some pressure to a girl to get what he wants sexually”; “I think that a girl who takes the initiative in sex is pushy”), and the remaining items directly assess the attitude of the respondent toward SDS (e.g., “I think sex is less important for girls than for boys”). The main limitation of this diversity of items is that the response will only predict the attitude toward the topic referred to in the items (i.e., double standards or certain sexual behaviors in men or women) (Ajzen and Fishbein, 2005).

### Social Norms and Assessment of Personal Adherence to the Sexual Double Standard

From a theoretical approach based on Social Cognition (Fiske and Taylor, 2013; Petty, 2018), we suggest that SDS research should involve a level of macropsychological analysis to explain personal adherence to SDS. This proposal is based on two assumptions. On the one hand, SDS is a social or collective phenomenon that influences the individual attitude of the people. On the other, the macropsychological and collective processes are different in nature from the psychological and individual ones (Páez and Campos, 2004; Wan et al., 2007). The phenomena of collective nature are composed of beliefs, emotions and behaviors referred to social groups and categories (Páez and Campos, 2004). Therefore, and in agreement with other authors (Wan et al., 2007), to obtain a macropsychological indicator of SDS it is convenient to measure the perception that the respondent has about which are the dominant attitudes that have the people with whom he/she interacts.

In this study, we propose a new measure that will serve to obtain a score about individual’s perception of societal SDS. The proposed measure is based on the SDSS (Muehlenhard and Quackenbush, 2011) and is designed to be applied in conjunction with another measure, also based on SDSS, that assesses the individual attitude of the respondent toward SDS (SDSS-S, Sierra et al., 2018). Next, we discuss the basis of this measure, and in what way it makes it possible to obtain a macropsychological indicator of the SDS.

The Theory of Planned Behavior (Ajzen, 1991) proposes that the intention of behavior is determined by the attitude toward that behavior and its subjective norm. Attitude refers to the personal evaluation toward a behavior (e.g., “I believe that a woman who takes the initiative in a sexual encounter is worthy of admiration” or “I feel contempt for a woman who takes the initiative”). The subjective norm refers to the person’s beliefs about what others think he/she should do (e.g., “Most people agree that a woman who takes the initiative in a sexual encounter is worthy of admiration”). The social norms have been pointed to as a cause of prejudice, “about half of all prejudiced attitudes are based only on the need to conform” (Allport, 1954). In addition, social norms determine the form that prejudice takes when expressing oneself. Gawronski and Bodenhausen (2011) found that implicit and explicit measures of prejudice against black people were more positively correlated with one another when subjects perceived that their subjective beliefs about black people were consistent with the perceived prejudice of society. According to these findings, individuals respond according to how they feel as long as they perceive that their environment validates their attitude (Crandall et al., 2002). Hence, it is necessary to assess not only individuals’ endorsement of the SDS but also their perception of society’s endorsement of the SDS (i.e., perception of societal SDS). The question is how to operationalize that measure.

To assess individuals’ endorsement of the SDS through standardized self-reports, subjects are asked to respond based on their own affective reactions and beliefs. This type of response is self-referred, that is, it refers to subjects’ personal endorsement of the SDS. By contrast, to assess subjects’ perception of societal SDS, responses should be hetero-referred, that is, the respondent uses society as a reference to make a judgment. In operational terms, respondents are asked to use their social environment as a reference to estimate society’s endorsement of the SDS.

### The Subjective Perception of the Collective Sexual Double Standard as a Macropsychological Indicator

Methodologists in Social Sciences have pointed out that analytical units obtained by aggregation of individual or micro level variables may be the easiest methodological and conceptual approach to model processes at the macro level (Coleman, 1986; Liska, 1990). According to this approach, we could obtain a macropsychological indicator of the SDS phenomenon by adding the responses to the self-referred measure of SDS validated by Sierra et al. (2018). However, we propose that the aggregation of responses to the hetero-referred measure will be a more valid indicator of how the SDS phenomenon works at a collective or macropsychological level (Kenny, 1996). How can we verify this supposed validity of the hetero-referred measure to evaluate SDS at the level of macropsychological analysis?

We should consider the following assumptions. First, the hetero-referred scale measures the SDS phenomenon as a macropsychological process; and the self-referred scale (SDSS-S; Sierra et al., 2018) measures the SDS phenomenon as a psychological process. Therefore, in terms of results, and since macro-psychological processes are different in nature to individual processes (Páez and Campos, 2004; Wan et al., 2007), the previous assumption is valid if the dimensions that capture both scales are different from each other. Note that research using standardized instruments to measure the SDS indicates that, although most young adults believe that there is SDS in society, individuals differ in their degree of endorsement (Milhausen and Herold, 2002).

Second, the phenomena of collective nature enjoy social consensus, and they fulfill functions that lead to adaptive consequences not only at a personal level, but at a social level (Páez and Campos, 2004). Therefore, to verify the validity of our assumption, we will test whether the responses of the individuals to the hetero-referred measure are related to their group membership (e.g., sex, education, age). Some authors consider that the SDS is a product of the patriarchal system that promotes male structural power (Sidanius and Pratto, 1999). Therefore, responses to the hetero-referred measure may be related to the group membership of the individuals to the social category of men or women. From the Theory of Social Identity (Tajfel and Turner, 1979), masculinity is a form of social identity that makes up the self-concept of oneself as a member of a social group. Social identity is conceived as a causal mechanism that intervenes in situations of social change (Tajfel, 1972); such change is observed, anticipated, desired or feared by the individuals involved (Tajfel, 1981). In this sense, individuals can determine or desire those sexual norms and behaviors that are most advantageous for the collective interests of the group with which they are identified (van Zomeren et al., 2008). From this approach, the SDS can be seen as an expression of individuals’ social motivation to maintain the differentiation of their group (i.e., ingroup) (Travis and White, 2000; Rudman et al., 2013). If the hetero-referred measure represents a level of macropsychological analysis of SDS, then it should capture the bias triggered by the social motivation to obtain advantages and privileges for the own group. From this framework, we assume that men, compared to women, will tend to underestimate the presence of traditional SDS in society. Likewise, although men show more personal adherence to SDS than women (Alison and Risman, 2013; England and Bearak, 2014; Sierra et al., 2018), we expect that in the hetero-referred measure men would have lower scores than women.

Moreover, system justification and social dominance theories suggest that individuals who justify social inequality are motivated to support ideologies of discrimination against women’s rights (Pratto et al., 1994) and accept traditional gender roles. From this approach, the SDS can be understood as the consequence of resistance to equality (Sidanius and Pratto, 1999). Consequently, the individuals who most defend inequality between social groups (Pratto et al., 1994), that is, people with a high orientation to dominance, will not only manifest greater personal adherence to the SDS (Sierra et al., 2018), but may also perceive little adherence to the traditional SDS by society in general (Brandt et al., 2015).

Higher levels of education has been negatively associated with personal endorsement of the SDS (Sierra et al., 2012, 2018), perhaps because more educated individuals are more sensitive to the asymmetry of sex roles in society. When the SDS was measured with the self-referred version (SDSS-S; Sierra et al., 2018), no significant difference was found among age groups, although older participants scored higher (Sierra et al., 2017). This may be because they have a greater motivation to maintain the asymmetric social norm and therefore minimize society’s endorsement of the SDS (Lott and Bullock, 2010). On the other hand, Spain initiated its democracy 40 years ago, and during that period the belief system and the norms on differences between both sexes have been progressively liberalized. In this sense, the generational socialization of the person can be a determining factor in their adherence to SDS. Within the cultural context of Spanish society there may be generational groups that adopt more or less traditional sexual scripts (Simon and Gagnon, 2003).

The SDSS (Muehlenhard and Quackenbush, 2011), is the most commonly used standardized instrument to assess SDS. This scale has recently been adapted to the Spanish heterosexual population (SDSS-S, Sierra et al., 2018). This version is a measure of the respondent’s attitude toward SDS and toward certain sexual behaviors in men and women (i.e., self-referred responses). The two-factor structure of the SDSS-S allows self-referred assessment of the subject’s attitude toward traditional and inverse SDS (i.e., inverse: SDS more in favor of women than men) in two areas: sexual freedom and sexual shyness. In this study, we propose a measure based on the original 26-item scale of the SDSS (Muehlenhard and Quackenbush, 2011), to assess the perception of societal SDS. This scale is intended to be a complementary measure to the self-referred version in order to understand the role of social norms of SDS on the personal support to SDS. The specific objective of this study was to explore the following features of the hetero-referred version of the SDSS: factor structure, internal consistency reliability, test–retest reliability, and some evidence of its validity.

We tested the following hypotheses:

H1. The macro-psychological processes are different in nature from the psychological and individual ones (Páez and Campos, 2004; Wan et al., 2007). We expect the SDSS-H will evaluate the collective norms regarding the SDS held by the Spanish society. Therefore, we expect a different multifactorial structure to the self-referred SDSS-S (Sierra et al., 2018).
H2. Items from both the SDSS-H and SDSS-S will allow us to evaluate different types of attitudes. That is, attitudes toward certain sexual behaviors in men or women, and attitudes toward the SDS in general. We expect higher correlations between the subscales of both the SDSS-H and SDSS-S that evaluate the same attitude (Crawford and Popp, 2003; Sakaluk and Milhausen, 2012).
H3. For individuals with a high social dominance orientation (SDO), we expect to also find that they:
  H3.1. Personally support traditional gender roles (SDSS-S) (Pratto et al., 1994; Sidanius and Pratto, 1999; Christopher and Wojda, 2008).
  H3.2. Underestimate social support of traditional gender roles (SDSS-H).
  H3.3. Overestimate social support toward structural changes in the traditional heteropatriarchy (inverse sexual double standard and/or social norm favorable to limit men’s sexual freedom) (SDSS-H).

We generally expect that the mean SDSS-H score will be more related to group membership based on gender, education and age (Coleman, 1986; Liska, 1990; Kenny, 1996). This assumption is specified in the following hypotheses.

H4. Men, *versus* women, underestimate the prevalence of the SDS in society (Travis and White, 2000; Rudman et al., 2013). When we measure the traditional SDS using the SDSS-H scale, we expect men’s score to be lower than women’s score.
H5. When we measure the traditional SDS using the SDSS-H scale, we expect the participants with a high level of education (i.e., university degree) will obtain higher scores than those with a lower level of education (e.g., middle or high school) (Sierra et al., 2012, 2018).
H6. When we measure the traditional SDS using the SDSS-H scale, we expect younger subjects (18–34 years) to obtain higher scores than older subjects (35–49, and 50 years or older) (Simon and Gagnon, 2003; Lott and Bullock, 2010; Sierra et al., 2017, 2018).

## Materials and Methods

### Participants

The sample was composed of 1,206 Spanish with heterosexual orientation. Inclusion criteria were: (a) being 18 or older and (b) heterosexual orientation. The latter criterion was established because, consistently with previous research (Marks and Fraley, 2005), and as recently defined in Zaikman and Marks (2017) “the sexual double standard (SDS) is the phenomenon whereby heterosexual men and women are evaluated differently regarding sex and sexuality” (p. 407). Also, items from the SDSS contain a greater depiction of traditional heterosexual scripts for both male and female. A convenience quota sampling method was used to obtain the same number of men and women. To depict all age ranges with a similar number of individuals distributed across them, we recruited participants from the following cohorts, used in previous Spanish research on sexuality and on the SDS Scale (see Sierra et al., 2018) and that allows an equivalent distribution: 18–34, 35–49, and 50 years old or older. In order to perform statistical analyses, the sample was randomly divided into two subsamples: Sample 1 (33.33%) and Sample 2 (66.67%). The sociodemographic information for each sample was as follows: (a) Sample 1: Range age 18–80, mean age 41.69 (*SD* = 13.49), most of them completed university studies (62.7%). Regarding some sexual-related information: their mean age at first sexual intercourse was 18.73 (*SD* = 3.53), mean number of sexual partners was 6.08 (*SD* = 11.01) and 80.85% was enrolled in a relationship at the time of the study from which 97.8% have sex with their partner; and (b) Sample 2: Range age 18–84, mean age 41.05 (*SD* = 14.62), most of them had university degree (66.6%). Regarding their sexuality, their mean age at first sexual intercourse was 18.42 (*SD* = 3.31), mean number of sexual partners was 5.22 (*SD* = 10.49) and 76% reported to be enrolled in a relationship, from which 97% have sex their current partner.

In order to estimate test–retest reliability, we incidentally selected a sample of 103 undergraduate students (85.4% women and 14.6% men) with a mean age of 19.17 years old (*SD* = 3.69). This group was compared to a sample of 126 undergraduate students who were randomly extracted from the total sample of 1,206 individuals. This group was composed of 75 women (59.5%) and 51 men (40.5%) with a mean age of 19.80 years (*SD* = 1.04). Both groups did not differ in relationship status (χ^2^_(1, 228)_ = 0.01, *p* = 0.906), age of first sexual intercourse (*t*_(193)_ = –0.12, *p* = 0.902) or number of sexual partners (*t*_(203)_ = 1.91, *p* = 0.058).

### Measurements

We designed a questionnaire on sociodemographic data and sexual history to obtain information about participants’ sex, age, nationality, education, sexual orientation (i.e., Which of these options better define your sexual orientation? Heterosexual, Same-sex orientation, Other in that case please specify), age of first sexual intercourse, number of sexual partners, relationship status, and sexual activity.

- Sexual Double Standard Scale (SDSS, Muehlenhard and Quackenbush, 2011). It consists of 26 items (e.g., “It’s worse for a woman to sleep around than it is for a man”) answered on a 4-point Likert scale ranging between 0 (disagree strongly), 1 (disagree mildly), 2 (agree mildly), and 3 (agree strongly). As indicated by Muñiz et al. (2013), a forward-translation of the items was conducted from English into Spanish by two researchers with adequate expertise in sexuality and English,- and a bilingual psychologist. Several modifications were performed based on their initial translation and adaptation, and also based on experts and a Spanish sample. Therefore, some experts evaluated the content comprehension and equivalence of each item, and finally a pilot study with a sample of Spanish undergraduate students was conducted. The sum of all items provides a global score. Higher scores indicate greater acceptance of the traditional SDS (i.e., greater sexual freedom for men). Regarding evidences of validity, the scale has shown associations with decreased sexual and relationship satisfaction for both men and women (Sánchez et al., 2005). Recently, among Spanish adolescents, the scale has shown associations with ambivalent sexism and sexist beliefs (Ubillos et al., 2016). A Cronbach’s alpha coefficient of 0.73 in women and 0.76 in men are reported (Muehlenhard and Quackenbush, 2011). In order to assess hetero-referred endorsement of the SDS, subjects received the following instructions to respond to the items: “We would like to know to what extent you believe that most people agree or disagree with each of the following statements.” The readers have an Supplementary Material with: the items in English and in Spanish of the SDSS-H, the final version in Spanish, and the instructions for its correction.

- Spanish version of Sexual Double Standard Scale (SDSS, Muehlenhard and Quackenbush, 2011; Sierra et al., 2018). This version is a self-referred measure of the sexual double standard (SDSS-S): subjects respond to it indicating to what extent they personally agree or disagree with the statements included in its items. It consists of 16 items, which are a translation into Spanish of the sixteen corresponding items of the original scale (Muehlenhard and Quackenbush, 2011), answered on a 4-point Likert scale ranging from 0 (disagree strongly) to 3 (agree strongly). Its two factors –Acceptance of sexual freedom (e.g., “It’s okay for a man to have sex with a woman he is not in love with”; ordinal Cronbach’s alpha = 0.84) and Acceptance of sexual shyness (e.g., “A woman who initiates sex is too aggressive”; ordinal Cronbach’s alpha = 0.87) – yield a Global Index of the SDS with adequate validity evidence. The score for each subscale is provided by the sum of their corresponding items. In Sample 2 of the present study, we found internal consistency values equal to 0.84 and 0.87 for Factor 1 and 2, respectively.

- Social Dominance Orientation Scale (SDOS, Pratto et al., 1994; Silván-Ferrero and Bustillos, 2007). Its 16 items (e.g., “Some groups of people are simply not the equals of others”) are answered on a 7-point Likert scale ranging from 1 (completely disagree) to 7 (completely agree). A total score can be obtained through the sum of the items. Higher scores indicate higher SDO. Silván-Ferrero and Bustillos (2007) demonstrated associations between the SDO, authoritarianism and gender. The reliability of the original scale was 0.91 (Pratto et al., 1994). This version has adequate reliability values (α = 0.85) (Silván-Ferrero and Bustillos, 2007). In Sample 2, Cronbach’s alpha was 0.78.

### Procedure

This study was approved by the Ethics Committee on Human Research of the University of Granada in Spain. The participants of this study did obtain written informed consent, which appeared on the first two pages of the questionnaire along with the battery of scales they were asked to respond. The SDSS (i.e., hetero-referred and self-referred) along with the informed consent form, the questionnaire on sociodemographic data and sexual history and SDO were administered by two evaluators in five universities from South and Central Spain (Granada, Almería, National Distance Education University, and Salamanca), and seven social centers and associations from Southern Spain. Participants answered the scales in an individual and private way. That is, participants completed the questionnaires in groups of approximately 30–40 or fewer in an available classroom in the described places, and they were sitting sufficiently far apart to ensure privacy. Once they had finished, they handed all the measures in a closed envelope. Some of the respondents, aged between 18 and 54, answered questionnaires available online. The URL of the questionnaires was distributed through the press and social media.

To calculate the test–retest reliability of the SDSS, the instrument was administered to university students in their respective classrooms at three different times by an expert researcher. In the first session, each participant was given three envelopes and three copies of the SDSS along with the informed consent form. All documents had the same code as well as the exact date of the second and third administration (at 4 and 8 weeks, respectively). After answering the scales, the students put them in a sealed envelope and delivered them to the evaluator.

## Data Analysis

In order to assess construct validity, the sample was randomly divided into two subsamples. Sample 1, composed of 402 individuals, was used to perform an exploratory factor analysis (EFA). Sample 2, composed of 804 individuals, was used to conduct a confirmatory factor analysis (CFA). The additional analyses were performed on the global sample. The EFA was performed with Factor 10.4 software. Thresholds for low factor loadings were >0.30 for the lower confidence interval. In case that an item had a cross loading, as far the difference was no higher than 0.15, no action was contemplated. We used the robust unweighted least squares (RULS) as an extraction method due to its performance with outliers and heteroscedastic errors (Midi et al., 2009). Normality distribution of data was not met (Mardias’ test = 63.53). The number of factors was explored with the optimal implementation of parallel analysis (PA) (Timmerman and Lorenzo-Seva, 2011) applied to the polychoric matrix. As we expected relationship between factors -confirmed *a posteriori* in the CFA- we decide to use an Oblimin rotation (Osborne, 2015). Confidence intervals were obtained by using Bootstrap in 500 samples. The CFA was conducted with EQS 6.1 software, also using a robust method (Maximum Likelihood, Robust; ML, R) applied again on the polychoric matrix. The overall fit indices used were the root mean square error of approximation (RMSEA) and its 90% confidence interval, as well as the comparative fit index (CFI) and the Bentler–Bonett Non-Normed Fit Index (NNFI). RMSEA values lower than 0.06 are indicative of good fit, and values lower than 0.08 indicate adequate fit (Hu and Bentler, 1999). CFI and NNFI values higher than 0.90 are considered as good fit, although ideally such values should be higher than 0.95 (Hu and Bentler, 1999). The Akaike Information Criterion (AIC, Akaike, 1974) was also taken, which indicates absence of FI if the increase with respect to the least restrictive model is considerable. The remaining analyses were performed with SPSS (Version 22.0) and R software. Missing data were handle with listwise deletion.

## Results

### Exploratory Factor Analysis

The PA indicated the existence of three factors (real-data% of variance was: 30.2, 17.6, 13.2, and 7.4 while the mean of random% of variance was: 11.8, 10.9, 10.1 and 9.3 furthermore the 95 percentile of random% of variance was 13.2, 12.0, 11.0 and 10.1). However, some items had low factor loadings and some factor loadings were distributed across several factors. For this reason, items 4, 5, 7, 8, 9, 10, 12, and 25 were eliminated and the EFA was performed again without modifying any parameters. Again, the PA yielded three factors that explained 56.63% of the variance. Table 1 shows the items and their factor loading distribution across the factors with their corresponding explained variances. In the lower limit and at a 95% confidence interval, all items had factor loadings greater than 0.30. Some items had lower (<0.40) communalities, this may be suggesting that the items does not really belong to that factor. But we will wait to see new suggestions in the further analysis.

**TABLE 1:** Exploratory factor analysis with Sample 1.

**Item**	**Acceptance of male sexual shyness**	**Acceptance of female sexual freedom**	**Acceptance of sexual double standard**	**Communalities**
1			**0.73 (0.65 to 0.80)**	0.57
2			**0.64 (0.53 to 0.71)**	0.48
3		**0.49 (0.34 to 0.61)**		0.30
6		**0.48 (0.33 to 0.60)**		0.35
11			**0.72 (0.64 to 0.78)**	0.62
13			**0.73 (0.65 to 0.80)**	0.60
14	**0.44 (0.30 to 0.56)**			0.22
15			**0.82 (0.76 to 0.87)**	0.70
16			**0.80 (0.73 to 0.86)**	0.78
17			**0.85 (0.78 to 0.91)**	0.73
18	**0.59 (0.43 to 0.70)**			0.39
19			**0.50 (0.37 to 0.58)**	0.32
20		0.30 (0.19 to 0.43)	**0.61 (0.52 to 0.69)**	0.51
21	**0.78 (0.68 to 0.88)**			0.64
22		**0.65 (0.54 to 0.76)**		0.55
23		**0.58 (0.39 to 0.69)**		0.34
24		**0.64 (0.50 to 0.75)**		0.44
26	**0.78 (0.68 to 0.86)**			0.61
% explained variance	29.28%	15.02%	12.33%	

*95% Confidence intervals are shown in brackets. Values under 0.30 in the lower limit of the confidence interval are omitted. Bold indicates items that are included in the final factor scores.*

### Psychometric Properties of the Items

Before attempting to confirm the factor structure obtained with the EFA, we assessed the psychometric properties of the items in order to better identify whether any item was undermining the scale. Table 2 shows that all ordinal alphas were higher than 0.70; the ordinal alpha would improve if item 14 was eliminated; however, that improvement was minimum (+0.02) and the item-total correlation was adequate, as was the case of the remaining items. For everything, finally item 14 was not deleted.

**TABLE 2:** Psychometric properties of the items.

**Item**	***M***	***SD***	**Kurtosis**	**Skewness**	**Corrected item-total correlation**	**Cronbach’s ordinal α if item deleted**	**Cronbach’s ordinal α**	**Total *M (SD*)**
	**Factor 1. Acceptance of male sexual shyness**							
14. I admire a man who is a virgin when he gets married	0.88	0.86	0.17	0.85	0.30	0.75		
18. I question the character of a man who has had a lot of sexual partners	1.04	0.82	–0.17	0.52	0.45	0.69	0.73	3.66 (2.29)
21. A guy who has sex on the first date is “easy”	0.93	0.86	–0.08	0.72	0.46	0.62		
26. A man who initiates sex is too aggressive	0.81	0.78	0.41	0.83	0.42	0.62		
	**Factor 2. Acceptance of female sexual freedom**							
3. It’s okay for a woman to have more than one sexual relationship at the same time	0.71	0.77	0.47	0.94	0.42	0.67		
6. I kind of admire a girl has had sex with a lot of guys	0.85	0.79	0.07	0.70	0.39	0.67		
22. It’s okay for a woman to have sex with a man she is not in love with	1.19	0.89	–0.64	0.33	0.48	0.62	0.70	5.22 (2.66)
23. A woman should be sexually experienced when she gets married	1.50	0.87	–0.69	–0.13	0.41	0.65		
24. It’s best for a girl to lose her virginity before she’s out of her teens	0.96	0.78	0.01	0.57	0.29	0.63		
	**Factor 3. Acceptance of sexual double standard**							
1. It’s worse for a woman to sleep around than it is for a man	1.53	1.08	–1.26	–0.13	0.67	0.88		
2. It’s best for a guy to lose his virginity before he’s out of his teens	1.45	0.91	–0.83	–0.00	0.52	0.89		
11. A woman who initiates sex is too aggressive	1.61	1.00	–0.103	–0.16	0.69	0.88		
13. I question the character of a woman who has a lot of sexual partners	1.62	0.95	–0.84	–0.38	0.68	0.88		
15. A man should be more sexually experienced than his wife	1.51	1.02	–1.12	–0.03	0.75	0.88	0.90	15.14 (6.33)
16. A girl who has sex on the first date is “easy”	1.87	1.01	–0.78	–0.54	0.72	0.88		
17. I kind of feel sorry for a 21-year-old man who is still virgin	1.61	1.04	–1.18	–0.11	0.46	0.88		
19. Women are naturally more monogamous – inclined to stick with one partner – than are men	1.93	0.88	–0.30	–0.58	0.49	0.90		
20. A man should be sexually experienced when he gets married	2.01	0.85	0.03	–0.72	0.44	0.89		

### Confirmatory Factor Analysis

The three-factor structure obtained previously showed adequate fit indices (see Table 3). However, all tridimensional models need 3 covariances between errors, as suggested by the Lagrange Multiplier test. These three pairs of items (1–2, 16–21, and 20–23) have a similar grammatical structure (e.g., item 20 “A man should be sexually experienced when he gets married” and item 23 “A man should be sexually experienced when he gets married”). Therefore, a similar distribution of their errors should be expected. Although none of the models reached optimal fit (RMSEA < 0.06, CFI > 0.95, NNFI > 0.95), they all reached acceptable fit. In fact, the model with three related factors showed the best fit. However, for theoretical reasons – the need to use a global score for the scale –, we decided to explore in greater detail the three-factor model with a second-order factor, as this model also yielded acceptable fit indices. Standardized weights can be observed in Table 4.

**TABLE 3:** Proposed models and model fit indices.

	**S-B χ^2^**	***df***	***p***	**RMSEA**	**RMSEA CI 90%**	**CFI**	**NNFI**	**AIC**	**χ^2^ difference test**
Unidimensional	1533.78	135	<0.01	0.114	0.108 to 0.119	0.830	0.809	1263.78	–
2 independent factors based on Sierra et al. (2018)	2148.71	100	<0.01	0.160	0.154 to 0.166	0.589	0.507	1948.71	–
2 related factors based on Sierra et al. (2018)	2679.11	101	<0.01	0.178	0.172 to 0.184	0.483	0.385	2477.11	–
3 independent factors	767.93	135	<0.01	0.076	0.071 to 0.082	0.924	0.913	497.93	–
3 independent factors with 3 covariances	624.29	132	<0.01	0.068	0.063 to 0.074	0.941	0.931	360.30	144.64^∗∗∗^
3 related factors	707.41	132	<0.01	0.074	0.068 to 0.079	0.931	0.920	443.41	–
3 related factors with 3 covariances	542.80	129	<0.01	0.063	0.058 to 0.069	0.950	0.941	284.80	164.61^∗∗∗^
3 factors with a second order factor	774.33	132	<0.01	0.078	0.073 to 0.083	0.923	0.910	510.33	231.53^∗∗∗^
3 factors with a second order factor and 3 covariances	623.37	129	<0.01	0.069	0.064 to 0.074	0.940	0.929	365.37	150.96^∗∗∗^

*S-B χ^2^ = is Satorra Bentler χ^2^; *df* = degrees of freedom; CFI = comparative fit index; RMSEA = root mean square error of approximation; CI RMSEA 90% = 90% confidence interval of the root mean square error of approximation; NNFI = bentler–bonett non-normed fit index; AIC = akaike’s information criterion; ^∗∗∗^*p* < 0.001.*

**TABLE 4:** Standardized loading weights, errors and explained variance for the 3 factors with a second order factor and 3 covariances model.

**Item**	**λ**	**Error**	***R*^2^**
i1	0.701	0.713	0.492
i2	0.500	0.866	0.250
i3	0.623	0.782	0.388
i6	0.552	0.834	0.305
i11	0.765	0.644	0.585
i13	0.753	0.658	0.567
i14	0.478	0.878	0.229
i15	0.791	0.612	0.625
i16	0.788	0.615	0.622
i17	0.680	0.733	0.462
i18	0.627	0.779	0.394
i19	0.605	0.796	0.366
i20	0.485	0.874	0.235
i21	0.668	0.744	0.446
i22	0.677	0.736	0.459
i23	0.559	0.829	0.312
i24	0.356	0.934	0.127
i26	0.629	0.777	0.396

### Test–Retest Reliability

To explore the temporal stability of this version, we used Pearson’s correlation coefficient. A 4-week period elapsed between the first time of data collection (T1) and the second time (T2); the third collection (T3) took place 8 weeks after T1. Overall, test–retest reliabilities were good for all three factors, with correlation values ranging from 0.44 to 0.79 (Table 5). We also used repeated-measures ANOVA to explore differences between the three time points. A multivariate analysis revealed no significant effect of time for either Factor 1 [*F*_(2, 91)_ = 0.57, *p* = 0.566], Factor 2 [*F*_(2, 91)_ = 2.75, *p* = 0.069] or Factor 3 [*F*_(2, 91)_ = 1.19, *p* = 0.307].

**TABLE 5:** Four and eight-week test–retest reliability of the SDSS – hetero-referred version.

	**Time 1–Time 2 (4 weeks)**	**Time 1–Time 3 (8 weeks)**
Factor 1. Acceptance of male sexual shyness	0.62^∗∗∗^	0.44^∗∗∗^
Factor 2. Acceptance of female sexual freedom	0.70^∗∗∗^	0.67^∗∗∗^
Factor 3. Acceptance of sexual double standard	0.79^∗∗∗^	0.64^∗∗∗^

**n* = 102 (Time 1), *n* = 98 (Time 2), *n* = 94 (Time 3). ^∗∗∗^*p* < 0.001.*

### Evidence of Validity

As for convergent validity, we calculated Pearson’s correlations between the following variables: Factor 1, Factor 2, and the Global Index of the self-referred version (SDSS-S); Factor 1, Factor 2, and the Global Index of the hetero-referred version (SDSS-H); and the total SDO score (see Table 6). As hypothesized, we found that the strength of the correlation between the self-referred version (SDSS-S) and the hetero-referred version (SDSS-H) was greater between similar attitude targets than between different attitude target. Factor 1 of the SDSS-S (i.e., Acceptance of sexual freedom) was correlated with Factor 2 of the SDSS-H (i.e., Acceptance of female sexual freedom) (*r* = 0.25, *p* < 0.001). Factor 2 of the SDSS-S (i.e., Acceptance of sexual shyness) and Factor 1 of the SDSS-H (i.e., male sexual shyness) were correlated (*r* = 0.42, *p* < 0.001), and the GI-SDS-S was significantly correlated with the GI-SDS-H (*r* = 0.13, *p* < 0.001). However, very low correlations were obtained between Factor 1 of the SDSS-S (i.e., Acceptance of sexual freedom) and the GI-SDS-H (*r* = 0.07, *p* < 0.05), between the GI-SDS-S and Factor 2 of the SDSS-H (i.e., Acceptance of female sexual freedom) (*r* = –0.07, *p* < 0.05); no correlations were found between the GI-SDS-S and Factor 1 of the SDSS-H. Altogether, H2 was supported. As hypothesized, the GI-SDS-S positively correlated with the SDO (*r* = 0.24, *p* < 0.001), which supports H3.1. Although scores on the GI-SDS-H were not significantly associated with SDO scores (H3.2), Factor 1 of the SDSS-H (i.e., Acceptance of male sexual shyness) was correlated with SDO (*r* = 0.18, *p* < 0.001), supporting H3.3.

**TABLE 6:** Zero-order correlations between the factors and global indices of the self-referred and the hetero-referred version of the SDSS and SDO.

	**1**	**2**	**3**	**4**	**5**	**6**	**7**
1. SDSS-S Factor 1. Acceptance of sexual freedom		–0.39^∗∗∗^	–0.12^∗∗^	0.25^∗∗∗^	–0.10^∗∗^	0.07^*^	–0.04
2. SDSS-S Factor 2. Acceptance of sexual shyness			0.42^∗∗∗^	0.01	0.23^∗∗∗^	–0.06	0.34^∗∗∗^
3. SDSS-H Factor 1. Acceptance of male sexual shyness				0.09^∗∗^	–0.04	–0.31^∗∗∗^	0.18^∗∗∗^
4. SDSS-H Factor 2. Acceptance of female sexual freedom					–0.07	–0.60^∗∗∗^	0.04
5. GI-SDS-S. Global Index of the Sexual Double Standard – Self-referred						0.13^∗∗∗^	0.24^∗∗∗^
6. GI-SDS-H. Global Index of the Sexual Double Standard – Hetero-referred							–0.04
7. SDO. Social dominance orientation							
*M*	11.25	6.36	3.64	4.24	0.47	30.01	43.20
*SD*	4.75	4.67	2.28	2.27	2.92	7.01	13.10
Possible range	0 to 24	0 to 24	0 to 12	0 to 12	–12 to 21	11 to 48	16 to 84
α	0.84	0.87	0.73	0.70	–	–	0.78

^∗∗∗^*p* < 0.001, ^∗∗^*p* < 0.01, ^*^p < 0.05.

We performed several MANOVAs to explore the differences in the mean scores of the Global Index of the SDS – Hetero-referred as a function of sex and education (Table 7). No significant differences were found in the GI-SDS-H according to sex (*F*_(1, 802)_ = 1.28; *p* = 0.257), thus H4 was not supported. Regarding education, significant differences were shown in the GI-SDS-H [*F*_(3,797)_ = 5.88; *p* = 0.001]; Bonferroni *post hoc* comparisons showed that scores on the GI-SDS-H were significantly higher in individuals with a university degree than in individuals with middle or high school; thus, H5 was supported. We also compared scores on the GI-SDS-H across age groups. To do so, we considered the following age groups: 18–34 years old, 35–49 years old, and 50 years old and older. We found significant differences in the Global Index [*F*_(2, 800)_ = 26.41; *p* = 0.000]. Bonferroni *post hoc* comparisons showed that scores on the GI-SDS-H were significantly higher in the youngest group (18–34 years old) than in the second and the third group (35–49 years old and 50 years or older); thus, H6 was supported (see Table 7).

**TABLE 7:** Means and standard deviations for the GI-SDS-H and GI-SDS-S according to sex, education and age groups.

	**GI-SDS-H**	**GI-SDS-S**
		
	***M (SD)***	***M (SD)***
**Sex**		
Male (*n* = 402)	33.55 (7.50)	1.27 (3.22)
Female (*n* = 402)	34.19 (8.50)	–0.32 (2.32)
**Education**		
Middle school (*n* = 79)	32.08 (7.08)	1.79 (3.95)
High school (*n* = 172)	32.43 (7.68)	0.68 (3.12)
University degree (*n* = 535)	34.68 (8.12)	0.22 (2.60)
**Age groups**		
18–34 (*n* = 267)	37.22 (8.28)	0.22 (2.68)
35–49 (*n* = 269)	32.34 (7.59)	0.37 (2.68)
50-older (*n* = 267)	32.03 (7.05)	0.81 (3.32)

*Range of scores for the GI-SDS-H is 0 to 54. Range of scores for the GI-SDS-S is –12 to +12.*

## Discussion

So far, empirical evidence shows difficulties for determining the prevalence of SDS (Gentry, 1998; Marks and Fraley, 2005). We consider a theoretical approach based on social cognition models (Fiske and Taylor, 2013), and a methodological one (Coleman, 1986; Liska, 1990; Kenny, 1996). We suggest that the prediction of individual support to SDS requires to bear in mind the role of the social norm about SDS. Therefore, we propose a hetero-referred version (SDSS-H) of the SDSS (Muehlenhard and Quackenbush, 2011), which aims to be a macropsychological indicator of the social norm in relation to the distribution of roles between men and women in the sexual behavior field.

The findings from the psychometric analyses show a three-factor structure that does not match the two-factor structure of the self-referred form (SDSS-S, Sierra et al., 2018). Also, we found that the orientation to social dominance is associated with different factors of both scales (i.e., SDSS-H and SDSS-S). We did not find differences in the average score of SDSS-H between men and women. However, differences were found in the response to SDSS-H when we compared social categories based on other criteria such as education or age. In particular, higher scores are found for individuals with the highest education (university degree) (vs. primary or secondary education) and for the youngest subjects (18–34 years) (vs. the oldest).

The main objective of this study was to examine the factor structure, reliability (internal consistency and test–retest reliability), and some evidences of validity of the SDSS-H in a sample of the heterosexual Spanish population. For this purpose, respondents were asked to use their social environment as a reference (i.e., hetero-referred responses) to estimate the endorsement they belief that society provides to SDS. The EFA showed a three factor structure. Once some items were deleted, this structure showed an adequate adjustment through CFA. In the first factor, items that assess the Acceptance of male sexual shyness are grouped; the second, comprised items that assess the Acceptance of women’s sexual freedom, and the third factor, measures the Acceptance of the traditional double sexual standard.

Out of the 26 original items from the SDSS, eight were eliminated due to their poor psychometric properties. It is likely that some topics depicted by these items, such as virginity for marriage, are considered as outdated. That is, for the Spanish Society of Contraception, where the average age of girls for first sexual intercourse is 16 years (Sociedad Española de Contracepción, 2016), the majority considers that virginity is no longer a requirement for marriage.

It should be noted that the factor structure of the SDSS-H does not match the structure of the self-referred version of the SDSS (SDSS-S, Sierra et al., 2018). We assume (Páez and Campos, 2004; Wan et al., 2007) that the SDSS-H measures the SDS at a macropsychological level of analysis, and whose nature is different from the level of individual analysis provided by the self-referred measure of SDS (SDSS-S, Sierra et al., 2018). We propose that both scales should be applied together when attempting to predict some types of sexual behavior as both will have a weight on the variance of the behavior in question. As pointed out, the main objective of this study is psychometric in nature. The intention here is not to demonstrate the predictive validity of both measures (SDSS-S and SDSS-H) on specific sexual behaviors. Further research is necessary to describe the predictive role of the social norm on SDS-related sexual behaviors.

However, when we are going to apply both scales together (SDSS-S and SDSS-H), the principle of compatibility must be taken into account (Ajzen and Fishbein, 2005). Specifically, the prediction of a behavior based on attitude requires that the indicators to measure the attitude imply exactly the same target to which the behavior to be predicted refers. Since the factors of the two scales, SDSS-S and SDSS-H, allow the evaluation of different targets, it is convenient to relate similar factors to predict compatible behaviors. In effect, the results support the H2. When, from each scale, we relate factors that imply the same attitude target, the correlations that result are higher.

The reliability was good for all three factors, as well as its temporal stability at 4 and 8 weeks. However, factor 1 (Acceptance for male sexual shyness) was the least stable. This may be due to individual factors related to the lack of individual’s willingness to accommodate to social change (Brandt et al., 2015). The fact that consistency progressively decreases across time, may be due to the interaction between the normative context and the respondent’s motivations to truly preserve the group distinctiveness (Falomir-Pichastor et al., 2013) or perhaps the traditional hetero-patriarchy structure. That is, factor 1 of SDSS-H prescribes a norm that threatens the traditional differentiation of sex roles for men and women. Consistently, recent research suggests that egalitarian norms may have the side effect of intensifying opposed-normative stances as egalitarianism threatens the desired distinctiveness (Falomir-Pichastor et al., 2017).

The relationship found between the individual characteristic (SDO) and the factors of both scales SDSS-S and SDSS-H led us to conclude that the results partially support H3. In agreement with previous findings (Pratto et al., 1994; Christopher and Wojda, 2008), the SDO is associated with an individual’s attitude toward the traditional SDS (SDSS-S, Sierra et al., 2018) (H3.1) (H3.1). Partially, our results support H3. Specifically, the SDO is associated with F1 of the SDSS-H (H3.3). That is, people who most support the structural differences between both genders in the sexual sphere are those who overestimate the support that society confers to undermine men’s sexual freedom. This finding indicates that the SDSS-H captures the tendency of those subjects with a high score in the SDO to “accept or reject ideologies and policies relevant to group relationships” (Pratto et al., 1994). More specifically, no significant relationship is found between SDO and F2 of the SDSS-H (acceptance of women’s sexual freedom) (H3.2). We believe that the issue of women’s sexual freedom, in Western societies, may be a target that is becoming reactive to differentiate between individuals with high and low adherence to SDS. We assume that in Western societies, the defense of sexual freedom, including the freedom of women, serves rather to differentiate who is modern from who is not, and logically most support sexual freedom. Another issue is, or is assumed to be, the defense of ideologies that support undermining men’s privileges. This is the case of Factor 1 of the SDSS-H. Therefore, it seems logical that a dispositional characteristic such as SDO, which predicts a subject’s inclination to maintain the *status quo* relation between genders, is significantly related to the ideologies that most threaten the traditional structural relationship of the heteropatriarchy.

We have assumed that the aggregation of individual responses in the SDSS-H measure will allow obtaining an evaluation of the collective norm in the field of sexual roles of both genders (Kenny, 1996). Therefore, we expect SDSS-H to be a more valid indicator of macropsychological processes than SDSS-S. Logically, we expected that scores on SDSS-H would be related to the individual’s group membership (Coleman, 1986; Liska, 1990; Kenny, 1996). Contrary to what was expected (H4) no differences are found for the responses for the hetero-referred version by gender. However, men report more personal adherence to SDS (Alison and Risman, 2013; England and Bearak, 2014) and higher scores in the SDSS-S than women (Sierra et al., 2018). There is evidence that beliefs related to the system (Jost et al., 2017) and sexism (Gómez-Berrocal et al., 2011; Monteith and Hildebrand, 2019) shape both individual and group perceptions of gender discrimination. Future research should examine in more depth the role of these processes in individual support for the SDS and in society’s perception of the SDS. As we expected (H5) individuals with greater academic degree (vs. middle or high school) obtained a higher average score in SDSS-H. Likewise, younger people (18–34 years) obtained significantly higher scores on SDSS-H than older people (35–49, and 50 years old or older) (H6). Taken together, it is likely that respondents with this demographic profile are more sensitive to the social norm favorable to SDS (Lott and Bullock, 2010). In contrast, other studies show that highly educated participants provide less support to SDS (Sierra et al., 2012, 2018); while older subjects (50 years old or older) (vs. subjects 18–34 years) indicate greater scores in the self-referred SDS measure (Sierra et al., 2017, 2018). Otherwise, the progressive sexual liberalization of Spanish society during the last four decades of democracy, perhaps has promoted less traditional sexual scripts that younger generations would have internalized to a greater extent (Simon and Gagnon, 2003).

## Limitations and Future Research Directions

Some limitations need to be mentioned. First, we cannot assure whether the order in which the self-referred and hetero-referred measure are administered may bias responses. Therefore, future research should check this. Second, research using experimental methodologies will clarify whether processes of social influence, social motivations and individual characteristics influence, alone or in mutual interaction, would account for the individual attitude of men and women toward SDS. In this sense, previous studies show the impact of cultural aspects such as religiosity on some forms of sexism (Hannover et al., 2018). Third, we consider that SDSS-H is a macropsychological indicator of SDS. Although this assumption was not straightly tested in our study we believe that some of our findings bring us closer to the idea that the level of analysis of SDSS-H is different from that of SDSS-S. Fourth, the application of our measure across countries would be very useful to validate its usefulness for capturing societal processes. Fifth, we have proposed that differences in SDSS-H by groups (e.g., education, age) may be due to the fact that this measure evaluates SDS at a macro level of analysis. A forthcoming investigation on the invariance of the measure will allow to establish more robust conclusions. Finally, it is necessary to test the measure in a representative sample, because the sample used in this study is incidental and composed, in a high percentage, of individuals with university studies.

## Conclusion

This research contributes to debate on the need to evaluate the SDS at different analysis levels (psychological-individual and macropsychological) to guarantee the prediction of sexual behaviors from the attitude someone expresses toward the SDS. Specifically, our work proposes measuring the perception of the SDS that exists in society. From a theoretical point of view, the proposal of our SDSS-H is based on the assumption that, in Western democratic societies, the form adopted by adherence to the SDS is inconsistent, since it is the result of the individual attitude that the person maintains, and of his perception of the social norm on that matter. In short, we assume that people’s attitude toward the SDS takes different forms due to the complacency and internalization processes of dominant social norm about this issue (Allport, 1954).

Previous research has been assumed that the progressive liberalism in heterosexual relationships may be the determinant of the inconsistency observed in adherence to the SDS by people from Western societies. Our approach suggests that this inconsistency may be due to a failure in the way the SDS is measured. This paper argues that a problem of evaluative inconsistency may occur when an attitude toward the SDS is measured through a questionnaire as it does not adequately predict the real behavior that men and women exhibit in sexual encounters. Making predictions only about the behaviors that are compatible with the target of the attitude being evaluated with a standardized instrument is recommended. As the SDSS-H is proposed to be used in conjunction with the SDSS-S, it is recommended that researchers comply with the principle of compatibility between the target being evaluated by both scales to interpret the results that derive from the joint application of both scales. The scales to which we refer herein (SDSS-S and SDSS-H) are constituted by factors that measure different targets. Finally, this measure provides several advantages and practical implications. On the one hand, like other forms of prejudice, the way in which people express their attitude toward SDS can be diverse. For example, it can be expressed in the form of a biased evaluation against women in the field of behaviors related to sexual shyness, but not in those related to sexual freedom. Such diversity in individual adherence to SDS may depend, at least in part, on processes of social influence. In other words, the tendency of the subject to be complacent with the social norm that exists on this issue may be determining a part of the variability observed in the prevalence of SDS. Since SDSS-H evaluates the social norm that a subject perceives about gender roles related to certain sexual behaviors, its joint use with the self-referred version, will allow to better predict the individual attitude toward SDS. On the other hand, by adding the responses to this hetero-referred measure, we can obtain a more valid macropsychological indicator to measure the SDS standard that exists in a given group. In this way, we can predict differences in SDS between groups and categories according to sex, age, educational level, culture or ethnicity.

## Data Availability

The datasets for this study can be found in the https://figshare.com/articles/Data_set_of_the_paper_A_proposal _of_a_societal_sexual_double_standard_measure_/7523138.

## Ethics Statement

This study was approved by the Ethics Committee on Human Research of the University of Granada in Spain. Written and informed consent was obtained from all participants.

## Author Contributions

MGB: conceptualization, formal analysis, project administration, resources, investigation, original draft preparation, writing, review and editing. PV-M: data curation, formal analysis, methodology, software, visualization, original draft preparation, writing, review and editing. NM: data curation, formal analysis, methodology, resources, investigation, original draft preparation, writing, review and editing. JS: conceptualization, funding acquisition, project administration, supervision, validation, original draft preparation, writing, review and editing.

## Conflict of Interest Statement

The authors declare that the research was conducted in the absence of any commercial or financial relationships that could be construed as a potential conflict of interest.
