# Supplementary material for: Sexual Double Standard: A Psychometric Study From a Macropsychological Perspective Among the Spanish Heterosexual Population
Source: Front Psychol. 2019 Aug 14;10:1869. doi: 10.3389/fpsyg.2019.01869 (PMC6702518; doi:10.3389/fpsyg.2019.01869)
Supplement: Supplementary file 1 [file Data_Sheet_1.docx]

***Supplementary Material***

**Supplementary Online Data**

**Items of SDSS-H in English and Spanish**

(1) It’s worse for a woman to sleep around than it is for a man (Es peor que una mujer se acueste con muchas personas a que lo haga un hombre)

(2) It’s best for a guy to lose his virginity before he’s out of his teens (Es mejor para un chico perder su virginidad antes de terminar la adolescencia que después)

(3) It’s okay for a woman to have more than one sexual relationship at the same time (Está bien que una mujer compagine más de una relación sexual al mismo tiempo)

(6) I kind of admire a girl who has had sex with a lot of guys (Siento cierta simpatía por una chica que ha tenido relaciones sexuales con muchos chicos)

(11) A woman who initiates sex is too aggresive (Una mujer que toma la iniciativa sexual es demasiado atrevida)

(13) I question the character of a woman who has had a lot of sexual partners (Pongo en duda el carácter de una mujer que ha tenido muchas parejas sexuales)

(14) I admire a man who is a virgin when he gets married (Admiro a los hombres que llegan vírgenes al matrimonio)

(15) A man should be more sexually experienced than his wife (Un hombre debe tener más experiencia sexual que su mujer)

(16) A girl who has sex on the first date is “easy” (Una chica que tiene relaciones sexuales en la primera cita es una chica “fácil”)

(17) I kind of feel sorry for a 21-year-old man who is still a virgin (Siento cierta lástima por un hombre que aún es virgen a los 21 años)

(18) I question the character of a man who has had a lot of sexual partners (Pongo en duda el carácter de un hombre que ha tenido muchas parejas sexuales)

(19) Women are naturally more monogamous –inclined to sitck with one partner- than are men (Las mujeres son por naturaleza más monógamas -propensas a permanecer con una misma pareja- que los hombres)

(20) A man should be sexually experienced when he gets married (Un hombre debería tener experiencia sexual antes de casarse)

(21) A guy who has sex on the first date is “easy” (Un chico que tiene relaciones sexuales en la primera cita es un chico “fácil”)

(22) It’s okay for a woman to have sex with a man she is not in love with (Está bien que una mujer tenga relaciones sexuales con un hombre del que no está enamorada)

(23) A woman should be sexually experienced when she gets married (Una mujer debería tener experiencia sexual antes de casarse)

(24) It’s best for a girl o lose her virginity befor she’s out of her teens (Es mejor para una chica perder su virginidad antes de terminar la adolescencia que después)

(26) A man who initiates sex is too aggressive (Un hombre que toma la iniciativa sexual es demasiado atrevido)

Note. Number of the item from the original scale appears in brackets.

**Spanish version of the SDSS-H**

A continuación aparece un listado de afirmaciones que acaba de contestar. Queremos conocer en qué grado cree usted que la mayoría de la gente está en desacuerdo o de acuerdo con lo que se afirma en cada una de ellas. Para indicar su respuesta utilice la escala numérica que aparece al lado de cada frase. Por favor, recuerde lo que significa cada número de la escala de respuesta

0= Muy en desacuerdo; 1= En desacuerdo; 2= De acuerdo; 3= Muy de acuerdo

1 Es peor que una mujer se acueste con muchas personas a que lo haga un hombre

2 Es mejor para un chico perder su virginidad antes de terminar la adolescencia que después

3 Está bien que una mujer compagine más de una relación sexual al mismo tiempo

4 Siento cierta simpatía por una chica que ha tenido relaciones sexuales con muchos chicos 0

5 Una mujer que toma la iniciativa sexual es demasiado atrevida

6 Pongo en duda el carácter de una mujer que ha tenido muchas parejas sexuales

7 Admiro a los hombres que llegan vírgenes al matrimonio

8 Un hombre debe tener más experiencia sexual que su mujer 0

9 Una chica que tiene relaciones sexuales en la primera cita es una chica “fácil”

10 Siento cierta lástima por un hombre que aún es virgen a los 21 años

11 Pongo en duda el carácter de un hombre que ha tenido muchas parejas sexuales

12 Las mujeres son por naturaleza más monógamas (propensas a permanecer con una misma pareja) que los hombres

13 Un hombre debería tener experiencia sexual antes de casarse

14 Un chico que tiene relaciones sexuales en la primera cita es un chico “fácil”

15 Está bien que una mujer tenga relaciones sexuales con un hombre del que no está enamorada

16 Una mujer debería tener experiencia sexual antes de casarse 0

17 Es mejor para una chica perder su virginidad antes de terminar la adolescencia que después

18 Un hombre que toma la iniciativa sexual es demasiado atrevido

Note. Number of the item from the original scale appears in brackets. Score from the Factor 1. Acceptance of male sexual shyness can be obtained by the following computation: item 14+item18+item 21+item 26, with scores ranging from 0 (Disagree strongly that most people accept male´s sexual shyness) to 12 (Agree strongly that most people accept male´s sexual shyness). Score from the Factor 2 Acceptance of female sexual freedom can be obtained by the following computation: item 3+item 6+item 22+item 23+ item 24, with scores ranging from 0 (Disagree strongly that most people accept women's sexual freedom) to 15 (Agree strongly that most people accept women's sexual freed). Score from the Factor 3. Acceptance of the traditional sexual double standard can be obtained by the following computation: item 1+ item 2+ item 11+ item 13+ item 15+ item 16 +item 17+ item 19+ item 20, with scores ranging from 0 (Disagree strongly that most people accept the traditional sexual double standard) to 27 (Agree strongly that most people accept the traditional sexual double standard). Score from the Global Index of the Sexual Double Standard – Hetero-referred (GI-SDS-H) can be obtained by the following computation Factor 1 (reverse score) + Factor 2 (reverse score) + Factor 3, with scores ranging from 0 (Disagree strongly that most people accept the traditional sexual double standard) to 54 (Agree strongly that most people accept the traditional sexual double standard).

| **Supplementary table indicating Pearson correlations between the items that correspond to both the SDSS-self reported version and the SDSS-hetero-refered version.** | | | | | | | | | | | | |  |
| --- | --- | --- | --- | --- | --- | --- | --- | --- | --- | --- | --- | --- | --- |
|  | SDSS-H3 | SDSS-H6 | SDSS-H11 | SDSS-H13 | SDSS-H14 | SDSS-H16 | SDSS-H18 | SDSS-H20 | SDSS-H21 | SDSS-H22 | SDSS-H23 | SDSS-H26 | |
| SDSS-S3 | .27*** | .05 | .11* | .08* | -.06 | -.00 | -.06 | .03 | -.15*** | .03 | .00 | -.05 | |
| SDSS-S6 | .16*** | .31*** | .08* | .00 | .00 | .02 | .00 | .08 | -.08* | .08* | .10** | .00 | |
| SDSS-S11 | .06 | .05 | .06 | .00 | .11** | .01 | .12*** | -.07 | .23*** | .03 | .03 | .16*** | |
| SDSS-S13 | .02 | .02 | .05 | .23*** | .09** | .13*** | .10*** | .04 | .25*** | -.03 | .01 | .13*** | |
| SDSS-S14 | -.06 | .04 | .01 | .05 | .37*** | .01 | .09** | -.04 | .11*** | -.08* | -.06 | .02 | |
| SDSS-S16 | -.06 | -.03 | .03 | .09** | .09** | .23*** | .14*** | -.02 | .38*** | -.05 | .00 | .13*** | |
| SDSS-S18 | .01 | -.00 | .01 | .17*** | .18*** | .08* | .29*** | -.02 | .30*** | -.04 | .03 | .19*** | |
| SDSS-S20 | .06 | .10** | -.05 | -.02 | -.09** | -.05 | -.02 | .28*** | .02 | .09* | .24*** | .06 | |
| SDSS-S21 | -.05 | .03 | .02 | .08* | .15*** | .18*** | .20*** | -.03 | .45*** | -.03 | .02 | .14*** | |
| SDSS-S22 | .14*** | .05 | .09* | .07* | -.11** | .02 | -.02 | .11** | -.13*** | .20*** | .08* | -.05 | |
| SDSS-S23 | -.02 | .09** | .12*** | -.09** | -.09** | -.11* | -.08* | .22*** | .22** | .11** | .32*** | .05 | |
| SDSS-S26 | .03 | .10** | .06 | .07* | .11*** | .02 | .20*** | .02 | .28*** | .04 | .10** | .27*** | |

*** *p* < .001; ** *p* < .01; * *p* < .05

**
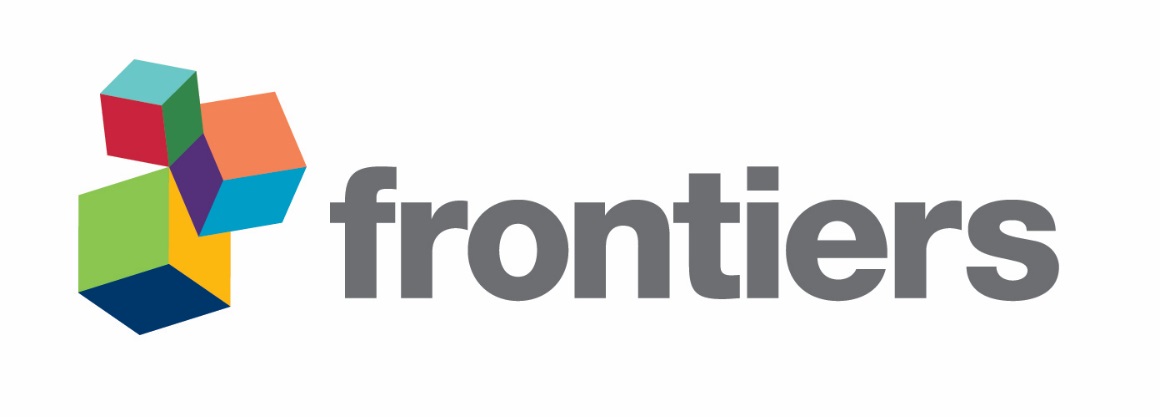
**
